# Photoprotective and antioxidant effects of Rhubarb: inhibitory action on tyrosinase and tyrosine kinase activities and TNF-α, IL-1α and α-MSH production in human melanocytes

**DOI:** 10.1186/1472-6882-13-49

**Published:** 2013-02-27

**Authors:** Jéssica PS Silveira, Leonardo N Seito, Samara Eberlin, Gustavo C Dieamant, Cecília Nogueira, Maria CV Pereda, Luiz C Di Stasi

**Affiliations:** 1 Laboratory of Phytomedicines, Department of Pharmacology, Institute of Biosciences, Universidade Estadual Paulista (UNESP), Botucatu, São Paulo CEP 18618-970, Brazil; 2 Research and Development Department of Chemyunion Chemistry Ltd, Av Independência 1501, Sorocaba, São Paulo CEP 18087-101, Brazil

**Keywords:** *Rheum rhaponticum*, Skin-lightening, Photoprotection, α-MSH, UV radiation, Melanocytes

## Abstract

**Background:**

Exposure to ultraviolet (UV) radiation causes various forms of acute and chronic skin damage, including immunosuppression, inflammation, premature aging and photodamage. Furthermore, it induces the generation of reactive oxygen species, produces proinflammatory cytokines and melanocyte-stimulating hormone (MSH) and increases tyrosinase activity. The aim of this study was to evaluate the potential photoprotective effects of *Rheum rhaponticum* L. rhizome extract on human UV-stimulated melanocytes.

**Methods:**

The effects of *Rheum rhaponticum* rhizome extract on tyrosine kinase activity, and on interleukin-1α (IL-1α), tumour necrosis factor α (TNF-α), and α-MSH production in human epidermal melanocytes were evaluated under UV-stimulated and non-stimulated conditions. Antioxidant activity was evaluated by lipid peroxidation and 1,1-dyphenyl-2-picryl-hydrazyl (DPPH) assays, while anti-tyrosinase activity was evaluated by the mushroom tyrosinase method.

**Results:**

*Rheum rhaponticum* L. rhizome extract showed *in vitro* antioxidant properties against lipid peroxidation, free radical scavenging and anti-tyrosinase activities, and inhibited the production of IL-1α, TNF-α, α-MSH, and tyrosine kinase activity in melanocytes subjected to UV radiation.

**Conclusions:**

These results support the inclusion of *Rheum rhaponticum* L. rhizome extract into cosmetic, sunscreen and skin care products for the prevention or reduction of photodamage.

## Background

Exposure to UV solar radiation has deleterious effects on human skin, inducing a number of skin disorders such as immune suppression, inflammation, photo-aging, sunburn, dermatitis, solar urticaria and skin cancer [1]. For protection against UV damage, human skin is equipped with a complex defence system, including skin pigmentation, skin thickening and a network of enzymatic and non-enzymatic antioxidants [2-4]. Skin pigmentation is one of the first events in response to UV radiation, which increases proliferation, activity and dendricity of melanocytes, followed by the synthesis and activation of tyrosinase and finally by the transfer of melanosomes to keratinocytes [2]. Melanocytes are key components of the skin pigmentary system and a significant source of various cytokines, including interleukin 1 α (IL-1α) and tumour necrosis factor α (TNF-α), which are released by UVB-exposed cells [2,5]. Skin pigmentation is also regulated by the activation of tyrosinase, a rate-limiting enzyme involved in the process of melanin synthesis (2). Interestingly, melanocyte growth and melanogenesis are also linked to the activation of tyrosine kinase activity and tyrosine kinase receptors [6,7]. In addition, the UV exposure of skin also results in significant oxidative stress via the generation of reactive oxygen and nitrogen species and is involved in the development of cutaneous inflammation [2,5]. These reactive species exert deleterious effects by oxidizing biologically essential molecules and induced oxidative damage of cellular membranes, tissues, and enzymes, which may lead to several diseases [4,8]. Oxidative stress is counteracted by the endogenous production of enzymatic antioxidants such as superoxide dismutase, glutathione peroxidase, and catalase and a large concentration of non-enzymatic antioxidants, mainly glutathione and exogenous components such as carotenoids, flavonoids, and other phenolic compounds, including vitamin E and vitamin C [1,3,9], which act by several mechanisms in the maintenance of health and prevention of several disorders and diseases [4,10]. On the basis of these observations, modulation of the metabolism of skin pigmentation by natural active compounds with anti-tyrosinase and antioxidant properties has been used as an innovative strategy to develop new products for skin photoprotection [3,11-14]. Although isolated plant compounds have a high potential in skin protection, whole herbs extracts like black tea, aloe and sesame oil showed better potential due to their chemical complex composition [15].

Plants from the Polygonaceae family that are rich in anti-tyrosinase and antioxidant stilbene compounds have been used as a source of photoprotectants and skin-lightening products [16-18]. These active stilbene compounds are plant secondary metabolites structurally related to resveratrol, a compound with well-documented potent photoprotective, antioxidant and anti-tyrosinase properties [19,20]. Recently, we demonstrated that another medicinal plant from the Polygonaceae family, *Coccoloba univefera*, protects human melanocytes after exposure to UV radiation [21]. On the basis of this evidence, *Rheum rhaponticum* L., an Asian medicinal plant commonly known as Rhubarb or Sibiric Rhubarb, was selected for the present study. In addition to its widely known laxative effect, this plant is also used to treat a broad spectrum of disorders, including kidney stones, gout, and liver and intestinal diseases [22]. Externally, this plant is also used to treat skin lesions such as sores and scabs [22]. In addition to containing stimulant laxative anthraquinones and astringents tannins, the rhizomes of *Rheum rhaponticum* and other polygonaceous species contain antioxidant and anti-tyrosinase hydroxystilbene compounds such as piecetannol, rhaponticin and desoxyrhaponticin [16-18].

Therefore, the aim of this study was to investigate the *in vitro* antioxidant and anti-tyrosinase activities of *Rheum rhaponticum* L. rhizome extract and its effect on the production of IL-1bα, TNF-α, α-MSH and tyrosine kinase activity in human melanocytes with or without exposure to UV radiation.

## Methods

### Plant material

*Rheum rhaponticum* L. dry rhizomes were obtained directly from Quimer Industries Ltd, São Paulo, Brazil. The voucher specimens are available within the supplier company where plant authentication was performed by botanists. Dry rhizomes were used to obtain a methanol extract, which was concentrated in a rotary evaporator. Plant extract was dissolved in an aqueous solution of 20% DMSO for antioxidant and anti-tyrosinase activities or in culture medium for studies on the irradiated and non-irradiated melanocyte cultures.

### Antioxidant and anti-tyrosinase activities

Two *in vitro* assays were used to test the antioxidant activity of the *Rheum rhaponticum* rhizome extract: the lipid peroxidation assay in rat brain membranes [23] modified from the original protocol [24] and the DPPH assay [25]. The plant concentration used for these assays ranged of 6 to 400 mg/ml. Quercetin, a potent antioxidant compound, was used as a reference drug. This experimental protocol met the “Guidelines of Animal Experimentation” and was approved by the Commission of Ethics in Animal Research (Protocol number 042/04-CEEA), Institute of Biosciences, Univ. Estadual Paulista, UNESP.

For anti-tyrosinase activity, enzymatic assay with mushroom tyrosinase [26] was used to evaluate tyrosinase inhibitory activity of the test compounds dissolved in 20% DMSO (50–3200 mg/ml). Kojic acid, a potent anti-tyrosinase agent, was used as a reference drug.

For both studies, each experiment was conducted in triplicate of six independent experiments.

### Cell culture and cell viability

Human epidermal melanocytes (Cryopreserved HEM, Cat. 104-05n) were subcultured in 25 cm^2^ flasks (Corning Inc, New York, NY) at 37°C in a 5% CO_2_ humidified incubator and expanded for at least five passages. After obtainment of the 80-90% confluence, cells were trypsinised and seeded using 24-well culture plates. After 24 hours, cells were washed with PBS, irradiated with different doses of ultraviolet radiation (75, 150, 300, 600 and 1200 mJ/cm^2^) and incubated with various doses (0.6 to 153.6 mg/mL) of plant extract. Cell-free supernatants and cell lysate were collected after 48 h of treatment, and assays were performed using commercial kits and conducted in triplicate of three independent experiments.

Cell viability was measured using the 3-(4,5-dimethylthi-azol-2-yl)-2,5-diphenyl tetrazolium bromide (MTT, Sigma) assay as described previously [27]. Melanocyte viability was evaluated after treatment with various plant extract concentrations and various UV doses. For UV irradiation was used a multiport solar UVA and UVB simulator (Model 601; Solar Light Co., Philadelphia, PA) equipped with a 150 W Xenon lamp, with two filters (a liquid and 1-mm Schott WG 320 filters) emitting a continuous spectrum of radiation beginning at 290 nm through the infrared spectrum and peaking at 400 nm. After this study the concentrations of 0.6, 1.2, 2.4 and 4.8 mg/ml and UV dose of 300 mJ/cm^2^ were selected because no loss of melanocyte viability was observed with these treatments.

### Quantification of biochemical mediators

The quantification of α-MSH was evaluated according methodology described in a commercial enzymatic immunoassay kit (Phoenix Pharmaceuticals Inc., Belmont, CA). α-MSH titres were expressed as ng/ml and were calculated by referring to standard curves constructed with known amounts of recombinant peptides.

The quantification of the IL-1α and TNF-α were were evaluated according methodology described in a commercial ELISA kit (DuoSet, R&D Systems, Minneapolis, MN). Cytokine titres were expressed as pg/ml and were calculated by referring to standard curves constructed with known amounts of recombinant cytokines.

An immunoprecipitation assay kit (US Biological, Swampscott, MA) was used to determine the tyrosine kinase activity. The observed tyrosine kinase activity was compared with known standards and expressed as pg/ml.

For all biochemical determinations, optical density was read using a microplate reader at 450 nm.

### Statistical analysis

For statistical analysis, one-way analysis of variance followed by the Tukey test was used to compare the data among all groups. Statistical significance was considered when *P* < 0.05.

## Results

### Antioxidant and antityrosinase activities

The effect of *Rheum rhaponticum* rhizome extract on the lipid peroxidation in rat brain membranes was compared with the effect of quercetin, a well-known flavonoid with potent antioxidant activity. The IC_50_ value of the extract was 8.92 ± 1.21 μg/ml, whereas the IC_50_ of quercetin was 0.53 ± 0.03 μg/ml. The EC_50_ scavenging activity of *Rheum rhaponticum* rhizome extract against DPPH radicals was 32.4 ± 4.37 μg/ml, whereas the EC_50_ of quercetin was 2.23 ± 0.82 μg/ml. The inhibitory effects of *Rheum rhaponticum* rhizome extract on tyrosinase activity were compared with those of kojic acid. The IC_50_ of the extract was 0.06 ± 0.01 μg/ml, and the IC_50_ of kojic acid was 0.02 ± 0.008 μg/ml.

### *Rheum rhaponticum* rhizome extract reduces α-MSH production in melanocytes

**Figure 1:**
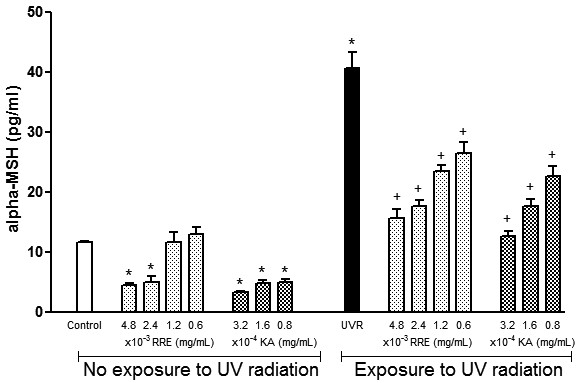
**Effects of *Rheum rhaponticum *L. extract (RRE) and kojic acid (KA) on alpha-melanocyte stimulating hormone (α-MSH) production by human melanocytes exposed and unexposed to ultraviolet radiation. **Data are presented as the mean ± S.E.M. of six individual experiments, performed in triplicate. *P < 0.001 compared with control unexposed to UV radiation; +P < 0.001 compared with control exposed to UV radiation.

α-MSH levels were assessed following exposure to *Rheum rhaponticum* rhizome extract. Reduced α-MSH release was elicited by the two highest doses (2.4 and 4.8 mg/ml) of *Rheum rhaponticum* rhizome extract (Figure 1). The treatment of melanocytes with UV radiation resulted in a 3.5-fold increase in α-MSH levels compared with non-irradiated melanocytes. Considering the groups that were exposed to UV radiation, a decrease in α-MSH levels was observed at all tested doses. These effects were similar to those produced by kojic acid.

### *Rheum rhaponticum* rhizome extract decreases the IL-1α level in melanocytes

**Figure 2:**
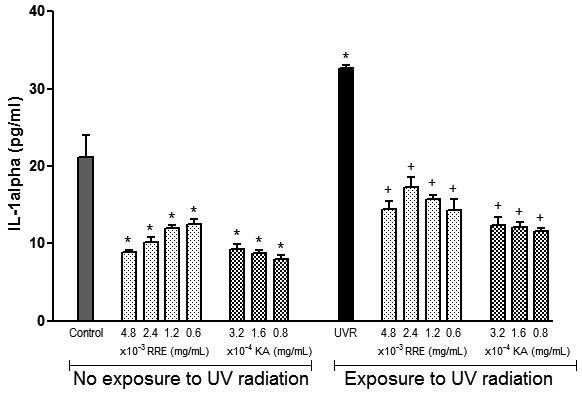
**Effects of *Rheum rhaponticum *L. extract (RRE) and kojic acid (KA) on interleukin-1 (IL-1) production by human melanocytes exposed and unexposed to ultraviolet radiation. **The data are presented as the mean ± S.E.M. of six individual experiments, performed in triplicate. *P < 0.001 compared with control unexposed to UV radiation; +P < 0.001 compared with control exposed to ultraviolet radiation.

IL-1α production was assayed in human melanocyte cultures that were and were not exposed to UV radiation (Figure 2). In the first set of experiments performed on melanocytes under basal conditions, *Rheum rhaponticum* rhizome extract decreased IL-1α levels at all tested doses compared with controls. This effect was similar to those produced by the reference compound, kojic acid. Treatment of melanocytes with UV radiation resulted in a 1.52-fold increase in IL-1α levels compared with controls. This increase was prevented similarly by both the plant extract and kojic acid.

### *Rheum rhaponticum* rhizome extract decreases the TNF-α level in melanocytes

**Figure 3:**
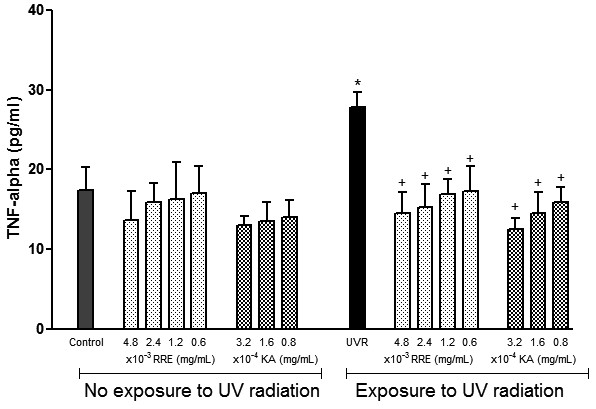
**Effects of *Rheum rhaponticum *L. extract (RRE) and kojic acid (KA) on tumour necrosis factor α (TNF-α) production by human melanocytes exposed and unexposed to ultraviolet radiation. **Data are presented as the mean ± S.E.M. of six individual experiments, performed in triplicate. *P < 0.001 compared with control unexposed to UV radiation; +P < 0.01 compared with control exposed to UV radiation.

The effects of *Rheum rhaponticum* rhizome extract on the TNF-α levels in melanocyte cultures were also evaluated under basal and irradiated conditions (Figure 3). TNF-α levels in melanocytes were not altered by the plant extract under basal conditions. Treatment of cells with UV radiation significantly increased TNF-α, which was prevented by the treatment of melanocytes with RRE. This inhibitory effect was similar to the inhibitory effect produced by the reference compound.

### Inhibitory effect of the *Rheum rhaponticum* rhizome extract on tyrosine kinase activity in melanocytes

**Figure 4:**
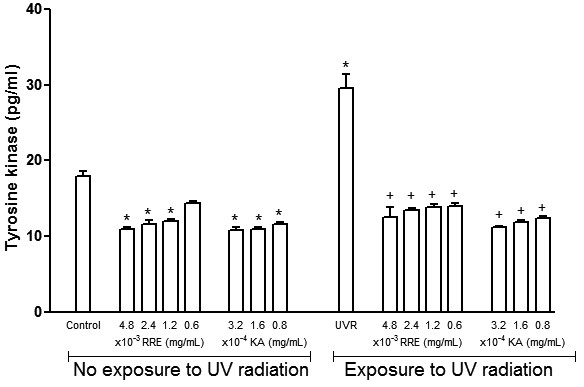
**Effects of *Rheum rhaponticum *L. extract (RRE) and kojic acid AKcp on tyrosine kinase activity in **h**uman melanocytes exposed and unexposed to ultraviolet radiation. **Data are presented as the mean ± S.E.M. of six individual experiments, performed in triplicate. *P < 0.001 compared with control unexposed to UV radiation; + P < 0.001 compared with control exposed to UV radiation.

*Rheum rhaponticum* rhizomes extract reduced tyrosine kinase activity in melanocyte cultures under basal conditions. As we expected, UV radiation produced a sharp 1.64-fold increase in enzymatic activity, which was also prevented by the treatment of cells with all concentrations of plant extract (Figure 4). This preventative effect was similar to that produced by the reference compound.

## Discussion

Natural compounds that originate from plants and plant extracts and are able to inhibit tyrosinase activity have been used as cosmetic additives and medicinal bioactive substances in skin-lightening and photoprotective products [2,11-15,28]. These natural products exhibit a range of important pharmacological activities to control hyperpigmentation and to produce UV skin protection acting as antioxidant, immune-modulatory and anti-inflammatory activities [2,9,14,29]. The skin pigmentation and tanning response to UV radiation is one of the most common examples of environmental adaptations in humans [30]. Pigmentary effects of UV radiation are mediated by pro-opiomelanocortin peptides (POMC), particularly α-MSH, which is produced by melanocytes and keratinocytes to regulate melanocytes and skin pigmentation [31-34]. It has been demonstrated that UV radiation increases the expression of the melanocortin-1 receptor which, after α-MSH agonist action, promotes a darkening effect [34,35]. In addition, α-MSH stimulates human melanocyte growth by binding to high-affinity receptors, particularly those related to tyrosine kinase [7]. Tyrosine kinase activation is also related to melanogenesis, and it has been demonstrated that responses produced by its activation were attenuated by tyrosine kinase inhibitors [36]. In our study, the data clearly showed that *Rheum rhaponticum* rhizome extract inhibits UV-induced α-MSH production in melanocytes. This inhibition also occurred under basal conditions and was similar to the pure reference compound, kojic acid, suggesting a potent effect for a crude plant extract. *Rheum rhaponticum* rhizome extract also inhibited tyrosine activity in human melanocytes under basal conditions and after UV stimulation. With the exception of *Coccoloba uvifera,* inhibitory effects on melanocyte α-MSH production have not been demonstrated for other polygonaceous plants or stilbene compounds [20,21,37]. However, medicinal plants from other botanical families have been shown to inhibit melanin biosynthesis via inhibition of α-MSH production in melanocytes [38-40].

Although the action of α-MSH is a key factor for skin pigmentation, studies have demonstrated that α-MSH also exhibits actions unrelated to pigmentation, such as secreting a range of molecules involved in skin homeostasis [34]. In fact, melanocytes have a complex network of functions and mediators; they can act as local stress sensors in the epidermis, as neuroendocrine cells, and as regulators of skin immune responses by producing several cytokines and other factors, including IL-1, TNF-α, IL-6, IL-3 and nitric oxide [34,41]. Interestingly, α-MSH also has potent anti-inflammatory and immune-modulatory activities because this peptide can antagonise the actions of pro-inflammatory cytokines and peroxide-generated oxidative stress [33,42]. In our study, the production of both IL-1α and TNF-α in UV-irradiated human melanocytes was increased compared with control cells. After incubation with all tested doses of plant extract, IL-1α and TNF-α production was significantly reduced. In addition, *Rheum rhaponticum* rhizome extract also reduced IL-1α and TNF-α production in non-irradiated human melanocytes.

The effects of α-MSH on melanogenesis are also mediated by tyrosinase, the rate-limiting enzyme in the process of melanin synthesis, which catalyses the necessary conversion of L-tyrosine to dopaquinone for the synthesis of both phaeomelanin and eumelanin. Thus, tyrosinase inhibitors are recognised as therapeutic products to treat local hyperpigmentation diseases and can be used as skin-lightening agents, such as resveratrol, kojic and ellagic acids, hydroquinone and arbutin [13,28]. In the present study, *Rheum rhaponticum* showed a potent anti-tyrosinase activity in the mushroom tyrosinase assay, similar to that produced by pure kojic acid. Although various natural compounds and plant extracts have been studied with the mushroom tyrosinase assay, *Rheum rhaponticum* rhizome extract inhibited activity by 50% at one of the lowest active doses that was tested compared with other natural products and plant extracts, including *Coccoloba uvifera*[11,21,25,43,44].

In addition to skin pigmentation, the decrease in reactive oxygen species generation has been shown to be important in other pathways involved in the prevention of UV-induced skin disorders [2,3]. Chronic exposure of skin to UV radiation results in an excessive production of reactive oxygen species, promoting oxidative stress and several skin-related disorders [45-47]. In fact, natural and synthetic antioxidant products have been studied as skin photoprotectants and skin-lightening agents, including polyphenols (flavonoids, coumarins, anthocyanins, catechins, tannins and stilbenes), alkaloids, isothiocyanates, carotenoids and vitamins [3,9,28,48,49]. Stilbene antioxidant compounds from the Polygonaceae family, including the *Rheum* species, have been linked to the prevention of skin photodamage [19,20,29,49]. These data are similar to those observed in our study, where the protective effects of *Rheum rhaponticum* rhizome extract was also linked to their antioxidant and radical scavenging properties, as evaluated by lipid peroxidation of brain membranes and DPPH assays, respectively. In addition, antioxidant capacity against lipid peroxidation and free radical scavenging capacity *in vitro* has been described as important assays to determine antioxidant properties of different compounds potentially useful to prevent a disease [10].

## Conclusion

Our short-term study provides the first evidence that *Rheum rhaponticum* rhizome extract can modulate damage induced by UV radiation. Because the skin response to UV radiation is a complex process, it might be argued that agents that contribute to re-establishing normal skin conditions should consist of as many mediators as possible. *Rheum rhaponticum* extract acts through multiple mechanisms, including antioxidant and radical scavenging properties, inhibition of tyrosinase and tyrosine kinase activities, and IL-1α, TNF-α and α-MSH production in human melanocytes subjected to UV stimulation. These results support the inclusion of this extract in skin-lightening products, as well as in cosmetic, sunscreen and skin care products for the prevention or reduction of photodamage. Clinical studies are necessary to support these claims, as is further elucidation of the pharmacological mechanisms involved.

## Competing interests

The authors declare that they have no financial and non-financial competing interests.

## Authors’ contributions

JPSS and LNS carried out the study, data collection and analysis. SE, GCD, CN and MCVP collaborated with plant extraction, cell culture studies and its data analysis. LCDS designed the study, supervised *in vitro* bioassays, biochemical evaluation and data analysis. All authors prepared the draft of manuscript. All authors read and approved the final manuscript.

## Pre-publication history

The pre-publication history for this paper can be accessed here:

http://www.biomedcentral.com/1472-6882/13/49/prepub
